# MRI findings in athletic groin pain: correlation of imaging with history and examination in symptomatic and asymptomatic athletes

**DOI:** 10.1007/s00256-024-04603-9

**Published:** 2024-02-02

**Authors:** Michelle Wei Xin Ooi, Matthew Marzetti, Emma Rowbotham, Dominic Bertham, Philip Robinson

**Affiliations:** 1 Musculoskeletal Centre X-Ray Department, Leeds Teaching Hospitals Trust, Chapel Allerton Hospital, Chapeltown Road, Leeds, LS7 4SA UK; 2 NIHR Leeds Biomedical Research Centre, Leeds Teaching Hospitals NHS Trust, Leeds, UK; 3 Department of Medical Physics, Leeds Teaching Hospitals NHS Trust, Leeds, UK

**Keywords:** Athlete, Elite, Professional, Groin pain, Core injury, Magnetic resonance imaging, Bone marrow oedema, Aponeurosis tear

## Abstract

**Objective:**

To determine differences in prevalence and diagnostic accuracy of MRI findings between asymptomatic athletes and athletes with longstanding groin pain.

**Materials and methods:**

One hundred twenty-three adult male athletes were approached with 85 consecutive athletes recruited. Group 1 (symptomatic, *n* = 34) athletes referred for longstanding groin pain (insidious onset, > 3 weeks duration). Group 2 (control, *n* = 51) athletes referred for injuries remote from the pelvis and no groin pain in the last 12 weeks. All referrers completed a clinical examination proforma documenting absence or presence of pelvis and hip abnormality. All patients completed the Copenhagen Hip and Groin Outcome Score (HAGOS) questionnaire and underwent a 3T MRI groin and hip protocol. MRIs were scored independently by two musculoskeletal radiologists blinded to clinical details. Statistical analysis was performed to evaluate associations between MRI findings, inter-reader reliability, clinical examination and HAGOS scores.

**Results:**

Pubic body subchondral bone oedema, capsule/aponeurosis junction tear and soft tissue oedema were more prevalent in the symptomatic group (*p* = 0.0003, 0.0273 and 0.0005, respectively) and in athletes with clinical abnormality at symphysis pubis, adductor insertion, rectus abdominis, psoas and inguinal canal (*p* = 0.0002, 0.0459 and 0.00002, respectively). Pubic body and subchondral oedema and capsule/aponeurosis tear and oedema significantly correlated with lower (worse) HAGOS scores (*p* = 0.004, 0.00009, 0.0004 and 0.002, respectively). Inter-reader reliability was excellent, 0.87 (range 0.58–1). Symphyseal bone spurring, disc protrusion and labral tears were highly prevalent in both groups.

**Conclusion:**

Clinical assessment and MRI findings of pubic subchondral bone oedema and capsule/aponeurosis abnormality appear to be the strongest correlators with longstanding groin pain.

**Supplementary Information:**

The online version contains supplementary material available at 10.1007/s00256-024-04603-9.

## Introduction

Athletes involved in running- and kicking-movement sports are commonly affected by longstanding groin pain [1, 2]. This is a complex clinical condition resulting in significant morbidity and is thought to be due to overloading of the symphysis pubis and parasymphyseal soft tissues [3, 4]. Establishing a clinical and radiological diagnosis can be difficult, given the complex pelvic anatomy with MRI, the preferred radiological investigation [5–8].

Previous clinical and radiology studies investigating groin pain in athletes have been cross-sectional or case-control studies of varying quality [9]. Many studies have used differing terminologies and clinical tests while imaging studies often do not include clinical assessment or reproducibility of findings [10, 11]. Several MRI findings have been described as abnormal including: degenerative changes at the pubic symphysis joint, adductor muscle origin enthesis pathology, pubic bone marrow oedema and the secondary cleft sign (short adductor attachment site tear) [12–15]. MRI studies have reported a higher prevalence of positive findings in symptomatic athletes (20–98%) compared with asymptomatic athletes (0–50%) [16–19]; however, some of the studies included around 50 subjects or less [16, 17]. Initially a number of MRI findings were thought to be specific for symptomatic athletes, but subsequent studies have shown that they can occur in asymptomatic athletes, for example pubic bone marrow oedema [16, 20–22]. Thus, more studies with a defined set of minimum reporting standards are required to validate the presence and severity of MRI findings in symptomatic and asymptomatic athletes [23].

This study aimed to evaluate the differences in prevalence and diagnostic accuracy of pelvic MRI findings between asymptomatic athletes and those with longstanding groin pain.

## Materials and methods

### Participants

**Fig. 1:**
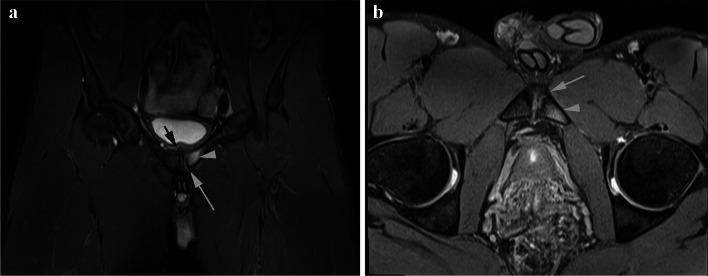
Symptomatic 24-year-old male athlete. **a** coronal STIR and **b** axial oblique PD-weighted fat suppressed MR images show left-sided pubic body (arrowhead) and subchondral bone (white arrow) marrow oedema. Right-sided bone marrow and bilateral capsule/aponeurosis soft tissue scored as normal. Symphysis pubis superior bone spurring (black arrow) scored as present

**Fig. 2:**
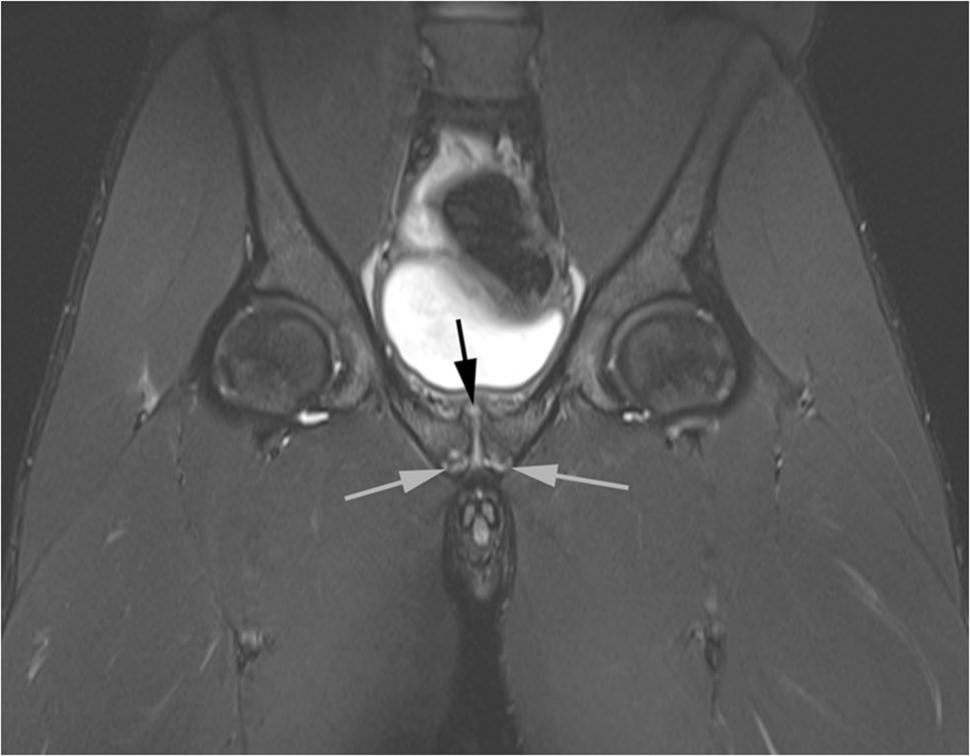
Control 18-year-old male athlete. Coronal STIR fat suppressed MR image shows bilateral subchondral bone marrow oedema (white arrows). Bilateral pubic body bone marrow signal and capsule/aponeurosis soft tissue scored as normal. Symphysis pubis superior bone spurring (black arrow) scored as present

This prospective study was approved by the institutional ethics review board and written informed consent was obtained from all participants. One hundred twenty-three adult male elite professional athletes were approached to participate in this study. Thirty-eight declined to participate in the study. A total of 85 consecutive adult male professional athletes were recruited into the study (mean age 24.0 years) and divided into symptomatic (*n* = 34, age range 17–35 years, mean age 23.5 years) and control (*n* = 51, age range 17–34 years, mean age 24.7 years) groups. The symptomatic group consisted of athletes referred by sports medicine clinicians for MRI for non-acute groin pain (insidious onset with more than 3 weeks duration) (Fig. 1). The control group was recruited from athletes referred for MRI of injuries remote from the anterior pelvis (including upper limb and lower limb below the knee) and with no history of athletic groin pain in the last 12 weeks (Fig. 2). None of the participants had any history of hip or groin surgery. None of the participants had any groin hernia on MR. For the 85 elite professional athletes recruited, sports performed were soccer (*n* = 52), rugby league (*n* = 22), cricket (*n* = 3), track athletics (*n* = 3) and boxing (*n* = 5).

### Patient reported outcome measures

All patients completed a standardised hip and groin questionnaire, the Copenhagen Hip and Groin Outcome Score (HAGOS) [24]. HAGOS covers six dimensions (subscales): symptoms, pain, function in activities of daily living (ADL), function in sport and recreation (Sports/Rec), participation in physical activities (PA) and quality of life (QOL). The six HAGOS subscales are scored separately: each question is scored from 0 to 4, where 0 indicates no problem. An aggregate score is not calculated as it is regarded desirable to analyse and interpret the different dimensions separately. Raw scores are then transformed to a 0–100 scale, with zero representing extreme hip and/or groin problems and 100 representing no hip and/or groin problems.

### Clinical assessment

All referrers (sports medicine clinicians) completed a clinical examination findings proforma where clinical tests were scored using a binary system blinded to MRI findings. These findings were evaluated on the right and left: symphysis pubis tenderness, adductor tenderness, adductor pain passive stretch, adductor pain resisted stretch, rectus abdominis tenderness, inguinal tenderness, psoas tenderness and hip rotation reduced.

### Imaging protocol

All patients underwent a standard MRI groin and hip protocol on a 3-T system (MAGNETOM Verio, Siemens Healthcare, Erlangen, Germany). For each MRI examination, coronal STIR and T1, axial T2 and sagittal T2 fat-saturated sequences of the entire pelvis were performed with the patient prone using an 18-channel body coil. Additionally, small field of view oblique axial through the symphysis pubis and sagittal PD sequences through both hips were performed using an 18-channel flexible surface coil. MR protocol and parameters for each sequence are detailed in Supplementary Table 1.

All MRI sequences were assessed independently by two MSK radiologists (author 3, 14 years experience and author 5, 24 years experience) blinded to the clinical details and images were scored (Supplementary Figures 1, 2, 3, 4)[22]:

1. Capsule/aponeurosis junction (defined as junction of the joint capsule, anterior pubic ligament, inguinal ligament and enthesis of adductor/rectus abdominis/pyramidalis tendons) assessed left and right for

- Soft tissue oedema, subjective score initially graded using a 4-point scale (0, normal; 1, mild; 2, moderate; 3, severe)
- Tear at tendon/capsular/enthesis junction scored 0–1 (0, absent or 1, present)

2. Pubic body assessed for bone marrow oedema (BMO) defined as

- Main body BMO > 2 cm, left and right subjective score relative to bone marrow signal in normal proximal superior pubic ramus initially graded using a 4-point scale (0, normal; 1, mild; 2, moderate; 3, severe)
- Subchondral BMO (the anteromedial pubic body)—left and right subjective score initially graded using a 4-point scale (0, normal; 1, mild; 2, moderate; 3, severe)
- Bone spurring/superior disc extrusion, presence on either side scored as 0–1 (0, absent or 1, present)

3. Hip region assessed for

- Hip acetabular labral tear, left and right scored 0–1 (0, absent or 1, present)
- Hip cartilage defect/osteochondral oedema, left and right scored 0–1 (0, absent or 1, present)
- Psoas tendinopathy/bursal fluid, left and right scored 0–1 (0, absent or 1, present)

### Statistical analysis

Statistical analyses was carried out using R version 4.3.0 [25]. The MRI scores for each anatomical area were evaluated for inter-reader reliability by Kappa analysis with agreement rated as follows: poor for kappa, < 0.21; moderate for kappa, 0.21–0.40; substantial for kappa 0.61–0.80 and excellent for kappa, >0.81 [26]. Where disagreement occurred, further analysis and discussion between the two readers were held to reach a consensus. Scores for abnormalities originally graded on a 4-point scale (e.g. oedema or tendinopathy) were simplified to normal (original grade 0 or 1) or abnormal (original grade 2 or 3) for statistical analysis and presentation [22, 27]. All clinical assessment and MRI scores were assessed, and the proportion of patients in each group with a positive score was calculated. The difference in proportions was calculated between each group, and proportion tests were used to calculate *p*-values. When sample size of the proportions was too small for proportions test, a binomial test was used. HAGOS scores were assessed for normality in each group using a Shapiro-Wilk test. As data was not normally distributed, a Mann-Whitney *U* test was used to compare HAGOS scores for each domain between groups. Finally, the calculated *p*-values were corrected using the Benjamini-Hochberg method to control the false discovery rate. All *p*-values quoted in this paper are corrected *p*-values unless otherwise specified.

## Results

### Patient reported outcome

All six domain HAGOS scores were consistently lower (worse) in the symptomatic group compared to the control group and significantly different between the two groups (Table 1). For example, mean score (SD) for function in sports and recreation domain was 52.9 (19.3) in the symptomatic group compared to control group 89.4 (14.5) with a *p*-value of less than 0.05 (Table 1).

**Table 1:** HAGOS scores

HAGOS	Total (*N* = 85)	Asymptomatic (*N* = 51)	Symptomatic (*N* =3 4)	Difference (median [95% CI])	*p*-value (corrected)
Quality of life					
Mean (SD)	70.5 (28.3)	87.9 (17.0)	44.3 (20.6)		
Median [Min, Q1, Q3, Max]	75.0 [10.0, 50.0, 100, 100]	95.0 [25.0, 77.5, 100, 100]	45.0 [10.0, 26.3, 58.8, 85.0]	−50 [−62.5–−36.7]	1.9 × 10^−10^
Physical activity					
Mean (SD)	72.2 (37.0)	93.1 (16.8)	40.8 (37.0)		
Median [Min, Q1, Q3, Max]	87.5 [0, 50.0, 100, 100]	100 [0, 93.8, 100, 100]	37.5 [0, 0, 75.0, 100]	−62.5 [−87.5–−50]	1.5 × 10^−9^
Sport and recreation					
Mean (SD)	74.8 (24.4)	89.4 (14.5)	52.9 (19.3)		
Median [Min, Q1, Q3, Max]	81.3 [12.5, 53.1, 100, 100]	96.9 [43.8, 84.4, 100, 100]	53.1 [12.5, 38.3, 71.1, 84.4]	−43.8 [−56.2–−35.9]	2.3 × 10^−10^
Activities of daily living					
Mean (SD)	88.7 (16.7)	95.5 (10.4)	78.5 (19.1)		
Median [Min, Q1, Q3, Max]	100 [30.0, 80.0, 100, 100]	100 [45.0, 97.5, 100, 100]	85.0 [30.0, 61.3, 93.8, 100]	−15.0 [−25–−10]	2.2 × 10^−6^
Pain					
Mean (SD)	87.1 (14.7)	95.6 (7.16)	74.3 (13.9)		
Median [Min, Q1, Q3, Max]	92.5 [42.5, 77.5, 100, 100]	100 [65.0, 95.0, 100, 100]	73.8 [42.5, 65.6, 84.4, 95.0]	−26.3 [−32.5–−20]	2.0 × 10^−10^
Symptoms					
Mean (SD)	76.0 (19.0)	86.3 (13.2)	60.5 (15.6)		
Median [Min, Q1, Q3, Max]	78.6 [25.0, 60.7, 92.9, 100]	89.3 [46.4, 78.6, 96.4, 100]	60.7 [25.0, 50.9, 70.5, 89.3]	−28.6 [−37.5–−21.4]	6.1 × 10^−9^

*Q1* 1st quartile, *Q3* 3rd quartile

### Clinical assessment

The referrer clinical examination scores showed positive findings for all variables in the control and symptomatic groups. All clinical tests showed a greater prevalence in symptomatic athletes than controls except for psoas tenderness (*p* = 0.1204) and reduced hip external rotation (*p* = 0.1911). The remaining clinical tests which all had *p*-values of less than 0.05 were symphysis pubis, rectus abdominis, adductor and inguinal tenderness, adductor pain on passive and resisted stretch and reduced hip internal rotation (Table 2).

**Table 2:** Clinical examination findings

Clinical examination findings	Total (*N* = 85)	Asymptomatic (*N* = 51)	Symptomatic (*N* = 34)	Difference [95% CI]	*p*-value (corrected)
Grouped clinical symptoms*					
0	45 (52.9%)	42 (82.4%)	3 (8.8%)		
1	40 (47.1%)	9 (17.6%)	31 (91.2%)	73.5% [56.9–90.1%]	1.5 × 10^-9^
Adductor resisted ± adductor passive					
0	45 (52.9%)	42 (82.4%)	3 (8.8%)		
1	40 (47.1%)	9 (17.6%)	31 (91.2%)	73.5% [56.9–90.1%]	1.5 × 10^−9^
Symphysis pubis					
0	55 (64.7%)	47 (92.2%)	8 (23.5%)		
1	30 (35.3%)	4 (7.8%)	26 (76.5%)	68.6% [50.1–87.1%]	3.6 × 10^−9^
Adductor insertion					
0	57 (67.1%)	47 (92.2%)	10 (29.4%)		
1	28 (32.9%)	4 (7.8%)	24 (70.6%)	62.7% [43.3–82.2%]	5.0 × 10^−8^
Adductor resisted					
0	57 (67.1%)	48 (94.1%)	9 (26.5%)		
1	28 (32.9%)	3 (5.9%)	25 (73.5%)	67.7% [49.0–86.2%]	3.6 × 10^−9^
Adductor passive					
0	64 (75.3%)	49 (96.1%)	15 (44.1%)		
1	21 (24.7%)	2 (3.9%)	19 (55.9%)	51.9% [32.0–71.9%]	1.4 × 10^−6^
Rectus abdominis					
0	70 (82.4%)	50 (98.0%)	20 (58.8%)		
1	15 (17.6%)	1 (2.0%)	14 (41.2%)	39.2% [19.8–58.6%]	6.5 × 10^−5^
Psoas					
0	73 (85.9%)	47 (92.2%)	26 (76.5%)		
1	12 (14.1%)	4 (7.8%)	8 (23.5%)	15.7% [−2.8–34.2%]	0.105
Inguinal tenderness					
0	72 (84.7%)	48 (94.1%)	24 (70.6%)		
1	13 (15.3%)	3 (5.9%)	10 (29.4%)	23.5% [4.5–42.6%]	1.6 × 10^−2^
Hip external					
0	72 (84.7%)	46 (90.2%)	26 (76.5%)		
1	13 (15.3%)	5 (9.8%)	8 (23.5%)	13.7% [−5.2–32.6%]	0.182
Hip internal					
0	71 (83.5%)	48 (94.1%)	23 (67.6%)		
1	14 (16.5%)	3 (5.9%)	11 (32.4%)	26.4% [7.0–45.9%]	7.2 × 10^−3^

*Q1* 1st quartile, *Q3* 3rd quartile

### Magnetic resonance imaging findings

MRI showed positive findings for all variables in control and symptomatic groups. There was no significant difference in the presence of bone spurs, disc extrusion, labral tear, hip cartilage defect, hip osteochondral oedema and iliopsoas abnormality between the symptomatic and control groups (Table 3).

**Table 3:** MRI findings

MRI findings	Total (*N* = 85)	Asymptomatic (*N* = 51)	Symptomatic (*N* = 34)	Difference [95% CI]	*p*-value (corrected)
Oedema main pubic body					
0	66 (77.6%)	44 (86.3%)	22 (64.7%)		
1	19 (22.4%)	7 (13.7%)	12 (35.3%)	21.7 % [0.4–042.6]	5.3 × 10^−2^
Subchondral pubic BMO					
0	50 (58.8%)	39 (76.5%)	11 (32.4%)		
1	35 (41.2%)	12 (23.5%)	23 (67.6%)	44.1 % [22.1–66.1 ]	3.6 × 10^−4^
Capsule/aponeurosis tear					
0	74 (87.1%)	48 (94.1%)	26 (76.5%)		
1	11 (12.9%)	3 (5.9%)	8 (23.5%)	17.6 % [2.0–33.3]	3.0 × 10^−2^
Capsule/aponeurosis oedema					
0	60 (70.6%)	44 (86.3%)	16 (47.1%)		
1	25 (29.4%)	7 (13.7%)	18 (52.9%)	39.2 % [17.5–60.9]	7.0 × 10^−4^
Bone spurs					
0	6 (7.1%)	5 (9.8 %)	1 (2.9 %)		
1	79 (92.9%)	46 (90.2 %)	33 (97.1%)	6.9 % [−3.1 %–16.8 %]	0.258
Disc extrusion					
0	7 (8.2 %)	6 (11.8 %)	1 (2.9 %)		
1	78 (91.8 %)	45 (88.2 %)	33 (97.1 %)	8.8 % [−1.7 %–19.3 %]	0.173
Labral tear					
0	56 (65.9%)	35 (68.6%)	21 (61.8%)		
1	29 (34.1%)	16 (31.4%)	13 (38.2%)	6.9 % [−16.3–30.0]	0.695
Acetabular subchondral oedema					
0	65 (76.5%)	40 (78.4%)	25 (73.5%)		
1	20 (23.5%)	11 (21.6%)	9 (26.5%)	4.9 % [−16.5–21.6]	0.805
Acetabular cartilage					
0	81 (95.3%)	49 (96.1%)	32 (94.1%)		
1	4 (4.7%)	2 (3.9%)	2 (5.9%)	2 % [−7.6–11.5]	0.695
Iliopsoas abnormality					
0	76 (89.4%)	47 (92.2%)	29 (85.3%)		
1	9 (10.6%)	4 (7.8%)	5 (14.7%)	6.9 % [−7.1–20.9]	0.353

*Q1* 1st quartile, *Q3* 3rd quartile

Three MRI variables showed a trend for presence in the symptomatic group compared to the control group with *p*-values of less than 0.05 (Table 3). These were pubic body subchondral BMO (*p* = 0.0003), capsule/aponeurosis junction tear (*p* = 0.0273) and capsule/aponeurosis junction soft tissue oedema (*p* = 0.0005). Pubic body BMO had an uncorrected *p*-value of 0.0382 (95% CI 0.4–42.6) but a corrected *p*-value of 0.0563.

### Magnetic resonance imaging versus patient reported outcome

Four MRI findings were significantly correlated with lower (worse) HAGOS scores for sports and recreation domain: pubic body BMO (*p* = 0.004), pubic subchondral BMO (*p* = 0.00009), capsule/aponeurosis tear (*p* = 0.0004) and capsule/aponeurosis oedema (*p* = 0.002) (Supplementary Tables 2–5).

The presence of pubic body BMO showed a trend for lower (worse) HAGOS scores in all domains except symptoms domain (Supplementary Table 2). Presence of pubic body subchondral BMO and capsule/aponeurosis junction tear showed a trend for worse HAGOS scores in all six domains (Supplementary Tables 3 and 4). Capsule/aponeurosis oedema showed a trend for worse HAGOS scores in all domains except physical activity (Supplementary Table 5).

Grouped MRI findings of pubic body BMO and/or subchondral BMO without capsule/aponeurosis junction tear or oedema were significantly correlated with lower HAGOS scores in four domains: quality of life (*p* = 0.034), physical activity (*p* = 0.028), sports and recreation (*p* = 0.031) and pain (*p* = 0.047).

Grouped MRI findings of capsule/aponeurosis oedema and/or tear without pubic body BMO or subchondral BMO were significantly correlated with lower HAGOS scores in five domains: quality of life (*p* = 0.013), sports and recreation (*p* = 0.006), activities of daily living (*p* = 0.032), pain (*p* = 0.015) and symptoms (0.022).

### Magnetic resonance imaging versus clinical assessment

For athletes with pubic body BMO on MRI, there was no significant difference between those with elicited tenderness in at least one of the following locations: symphysis pubis, adductor insertion, rectus abdominis, psoas and inguinal canal compared to those without (*p* = 0.447).

Three MRI variables were found to be more prevalent in athletes with elicited tenderness in at least one of the above listed locations compared to those without. These MRI variables were pubic body subchondral BMO (*p* = 0.0002), capsule/aponeurosis tear (*p* = 0.0459) and capsule/aponeurosis soft tissue oedema (*p* = 0.00002).

Grouped MRI findings of pubic body BMO and/or subchondral BMO without capsule/aponeurosis junction tear and/or oedema were not found to be significantly correlated with elicited tenderness in at least one of the above listed locations (corrected *p*-value = 0.0603) although its uncorrected *p*-value was 0.0463.

Grouped MRI findings of capsule/aponeurosis oedema and/or tear without pubic body BMO and/or subchondral BMO were found to be associated with elicited tenderness in at least one of the above listed locations (*p*-value = 0.001).

### MRI inter-reader reliability

The overall inter-reader reliability was excellent, Kappa = 0.87 (range 0.58–1) (Supplementary Table 6).

## Discussion

This study revealed that certain MRI findings, commonly thought to be abnormal, also occur in control patients and patients with normal clinical examination findings. However, some MRI findings showed a stronger association with clinical and symptom scores.

### Pubic body bone marrow oedema

Pubic body bone marrow oedema (BMO) was not found to be more prevalent in the symptomatic athlete group compared to control group in our study. Additionally, moderate to severe pubic body BMO was present in athletes with normal clinical findings. This is in contrast to some previous studies which found that symptomatic athletes were more likely to have moderate to severe pubic BMO when compared to control groups consisting of a mixture of asymptomatic soccer and non-soccer athletes, umpires and non-athletes [19, 28]. Previous systematic reviews have found mixed results on the significance of pubic body BMO in athletes with groin pain and concluded that better quality studies were needed with more relevant controls [12, 14]. A recent study with a more relevant control group, consisting of asymptomatic athletes involved in cutting-and-kicking movement sports, found no significant difference in pubic body BMO when compared to symptomatic athletes with groin pain [29], concurring with our study result. We note that the uncorrected *p*-value for moderate/severe pubic body BMO in our study was initially less than 0.05 but increased to 0.053 after correction and was therefore considered statistically not significant. This could be due to the study being underpowered as the sample size was smaller than planned due to the Covid-19 pandemic. Our study result could also vary from previous studies given our control group were athletes involved in cutting-and-kicking movement sports and exposed to pelvic loading [30]. This could also explain why findings such as symphysis pubis bone spurring and disc extrusion were highly prevalent in both patient groups, simply representing changes related to chronic overload.

### Pubic body subchondral oedema

Pubic body anteromedial subchondral BMO was found to be significantly more prevalent in the symptomatic group and significantly associated with lower HAGOS scores in all domains. Athletes with elicited tenderness in at least one of the following locations, symphysis pubis, adductor insertion, rectus abdominis, psoas or inguinal canal, were also more likely to have pubic body subchondral oedema. This anteromedial region of the pubic body represents an area of marked anatomical transition where the pubic apophysis forms and is immediately adjacent to the capsule and merging anterior aponeurosis. This focal part of the pubic bone has not been formally assessed in previous studies. Oedema may be more clinically relevant to symptoms, pain perception and positive clinical findings because it represents focal overload of the old growth plate region and adjacent soft tissues. Previous studies have found that the periosteum is the most densely innervated tissue of the bone and hypothesised that bone pain is generally perceived as sharper and more focal when the periosteum is involved [31–34]. This could also explain our study findings of moderate to severe pubic subchondral BMO being strongly associated with clinical symptoms and tenderness on examination while pubic body BMO had mixed results as detailed above.

### Capsule/aponeurosis tear and soft tissue oedema

**Fig. 3:**
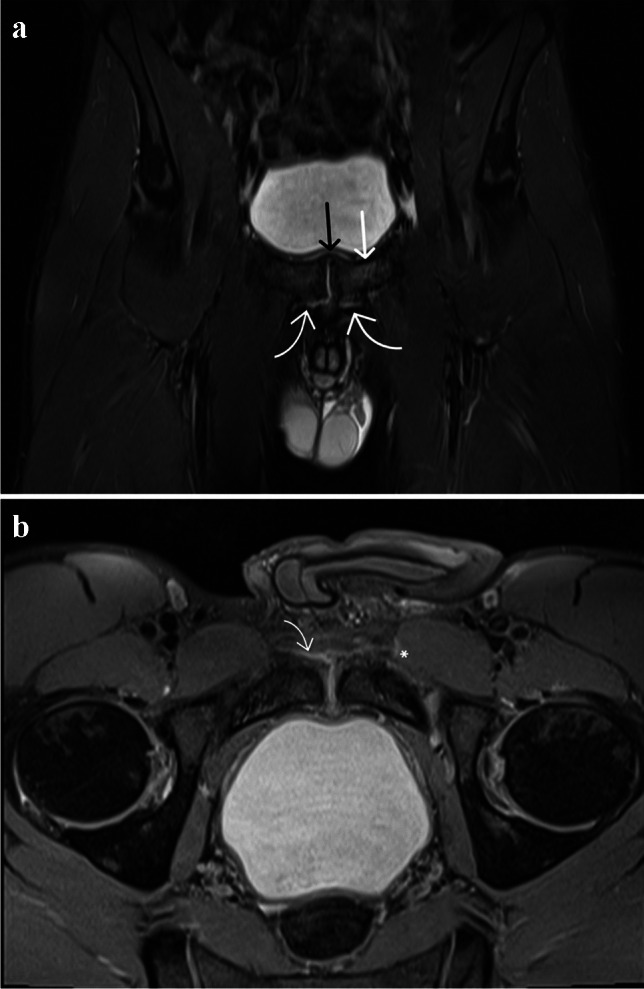
Symptomatic 27-year-old male athlete. **a** coronal STIR and **b** axial oblique PD-weighted fat suppressed MR images show left-sided pubic body marrow oedema scored as grade 2 (white straight arrow), bilateral capsule/aponeurosis tears (curved arrows) and left capsule/aponeurosis soft tissue oedema (asterisk). Right capsule/aponeurosis soft tissue oedema was present but not shown on above images. The right pubic body marrow signal was scored grade 1, therefore categorised as normal. Symphysis pubis superior bone spurring (black arrow) scored as present

Capsule/aponeurosis tear and soft tissue oedema was significantly more prevalent in the symptomatic group, significantly associated with lower HAGOS scores in all domains and with tenderness in at least one of the following locations: symphysis pubis, adductor insertion, rectus abdominis, psoas or inguinal canal Fig. 3. This is comparable to previous studies which showed a strong association between the presence of a cleft sign (capsule/aponeurosis tear) and groin pain [15, 18]. A previous study also showed that capsule/aponeurosis tear was associated with a delayed return-to-play time in athletes with groin pain compared to those without capsule/aponeurosis (24.7 weeks vs 11.9 weeks median time to return-to-play) [35].

### Grouped MRI findings vs patient reported outcome

Pubic body BMO and/or subchondral BMO as well as capsule/aponeurosis tear and/or oedema were independent factors for worse HAGOS scores in pain, symptoms and sport and recreation. This would suggest that these MRI variables could be an indicator for a degree of functional restriction in sport activities. However, it is important to note that the HAGOS score was designed to assess symptoms and activity limitations in active young to middle-aged patients with long standing hip or groin pain [24]. Its relation to elite athletic performance remains unclear, but it remains the best available patient outcome scoring system given no other comparable athlete specific questionnaire tools have been made available and tested during the time of this study.

### Grouped MRI findings vs clinical assessment

Athletes with capsule/aponeurosis tear and/or oedema without any pubic bone oedema were more likely to have symphysis pubis, adductor insertion, rectus abdominis, psoas and/or inguinal tenderness. In contrast, pubic bone oedema without any capsule/aponeurosis abnormality was not significantly associated with tenderness in the above listed locations on clinical assessment. This suggests that capsule/aponeurosis abnormalities tend to be more symptomatic compared to pubic BMO which would support previous theories that pubic body BMO can occur as a result of chronic overuse or subclinical injury [17, 21, 28].

### Hip and iliopsoas

There was no significant difference in the presence of a labral tear, acetabular cartilage defect or iliopsoas abnormality between symptomatic and control groups. This is in keeping with multiple previous studies which have highlighted that labral tears are a common finding on MRI, and careful correlation clinically is needed before attributing cause of symptoms to a labral tear [27, 36–38].

## Strengths and limitations

The sample size obtained in this study was smaller than planned (180 originally planned with 90 in each group) due to the Covid-19 pandemic which led to a reduced referral rate for both patient groups. Cam and pincer morphology was not specifically evaluated during this study given controversies in its clinical significance [39] and was beyond the scope of the current study. The authors did however study the presence of labral tear and cartilage damage which are potential surgical targets at our institution [40]. Strengths of this study include prospective data collection, clinical symptom and examination data documented, use of a standardised MRI protocol, inter-reader testing and blinded review by two experienced musculoskeletal radiologists. Another strength was the use of elite athletes in sports prone to groin pain in the control group.

## Conclusion

Symptomatic athletes were more likely to have moderate to marked pubic body subchondral BMO, capsule/aponeurosis tear and soft tissue oedema. Clinical findings can be positive in asymptomatic athletes, but there is a trend for these to be more prevalent in symptomatic athletes. HAGOS scores were reduced in symptomatic athletes but also in any athlete with moderate/severe pubic body BMO, subchondral BMO, capsule/aponeurosis junction tear or soft tissue oedema on MRI irrespective of their current symptoms. The findings of this study show the complexity and difficulty in interpretation of clinical and imaging findings in these athletes. MRI findings can be non-specific in athletes involved in sports where training and participation leads to anterior pelvis loading. Clinical assessment along with MRI findings relating to moderate to marked pubic body subchondral BMO and capsule/aponeurosis abnormality seem to be the strongest correlators to consider when assessing athletes with longstanding athletic groin pain.

## Supplementary information

ESM 1 (DOCX 66 kb)

ESM 2 (TIF 232 kb)

ESM 3 (TIF 257 kb)

ESM 4 (TIF 371 kb)

ESM 5 (TIF 368 kb)
