# Supplementary material for: MRI findings in athletic groin pain: correlation of imaging with history and examination in symptomatic and asymptomatic athletes
Source: Skeletal Radiol. 2024 Feb 2;54(4):841–50. doi: 10.1007/s00256-024-04603-9 (PMC11845425; doi:10.1007/s00256-024-04603-9)
Supplement: Supplementary file 1 — (DOCX 66 kb) [file 256_2024_4603_MOESM1_ESM.docx]

**Supplementary Material**

Supplementary Table 1. MRI Pelvis Imaging Protocol

| Plane/Sequence | FOV (mm) | Voxel size (mm³) | TR (ms) | TE (ms) | TI (ms) |
| --- | --- | --- | --- | --- | --- |
| Coronal STIR | 380 | 1.0×1.0×4.0 | 5410 | 50 | 160 |
| Coronal T1 TSE | 380 | 0.5×0.5×4.0 | 700 | 21 | - |
| Axial T2 TSE | 380 | 0.8×0.8×3.0 | 5870 | 73 | - |
| Sagittal T2 fat-saturated TSE | 180 | 0.7×0.7×3.0 | 3000 | 70 | - |
| Oblique axial PD TSE through symphysis pubis* | 170 | 0.5×0.5×3.0 | 3200 | 73 | - |
| Sagittal PD TSE through both hips | 180 | 0.6×0.6×3.0 | 3750 | 24 | - |

*****parallel to superior aspect of symphysis pubis and superior pubic rami

Supplementary Table 2. Pubic Body Bone Marrow Oedema vs HAGOS

|  |  | Pubic Body Bone Marrow Oedema | |  |  |
| --- | --- | --- | --- | --- | --- |
|  | Total (N=85) | 0 (N=66) | 1 (N=19) | Difference (95% CI) | p-value (corrected) |
| **Quality of life** |  |  |  |  |  |
| Mean (SD) | 70.5 (28.3) | 74.5 (27.1) | 56.3 (28.5) |  |  |
| Median [Min, Q1, Q3, Max] | 75.0 [10.0, 50.0, 100, 100] | 85.0 [10.0, 55.0, 100, 100] | 60.0 [10.0, 35.0, 77.5, 100] | - 25 [-50 - -5] | 2.3x10^-2^ |
| **Physical activity** |  |  |  |  |  |
| Mean (SD) | 72.2 (37.0) | 78.0 (34.8) | 52.0 (38.2) |  |  |
| Median [Min, Q1, Q3, Max] | 87.5 [0, 50.0, 100, 100] | 100 [0, 75.0, 100, 100] | 37.5 [0, 25.0, 93.8, 100] | -62.5 [-87.5 - -62.5] | 1.6x10^-2^ |
| **Sport and recreation** |  |  |  |  |  |
| Mean (SD) | 74.8 (24.4) | 79.6 (21.4) | 58.2 (27.4) |  |  |
| Median [Min, Q1, Q3, Max] | 81.3 [12.5, 53.1, 100, 100] | 84.4 [25.0, 66.4, 100, 100] | 53.1 [12.5, 39.1, 87.5, 100] | -31.25 [-46.9 - 3.1] | 4.4x10^-3^ |
|  |  |  |  |  |  |
| **Activities of daily living** |  |  |  |  |  |
| Mean (SD) | 88.7 (16.7) | 91.5 (13.5) | 78.9 (22.6) |  |  |
| Median [Min, Q1, Q3, Max] | 100 [30.0, 80.0, 100, 100] | 100 [45.0, 86.3, 100, 100] | 85.0 [30.0, 60.0, 100, 100] | -15[-40 - -15] | 4.0x10^-2^ |
| **Pain** |  |  |  |  |  |
| Mean (SD) | 87.1 (14.7) | 89.5 (12.5) | 78.6 (18.8) |  |  |
| Median [Min, Q1, Q3, Max] | 92.5 [42.5, 77.5, 100, 100] | 95.0 [45.0, 82.5, 100, 100] | 80.0 [42.5, 65.0, 96.3, 100] | - 15 [-30 – 1.25] | 3.3x10^-2^ |
| **Symptoms** |  |  |  |  |  |
| Mean (SD) | 76.0 (19.0) | 78.2 (18.1) | 68.0 (20.4) |  |  |
| Median [Min, Q1, Q3, Max] | 78.6 [25.0, 60.7, 92.9, 100] | 82.1 [25.0, 65.2, 92.9, 100] | 64.3 [39.3, 51.8, 82.1, 100] | -17.9 [-31.9 – 3.6] | 5.7x10^-2^ |
| Q1 = 1st quartile Q3 = 3rd quartile | | | | | |

Supplementary Table 3. Subchondral Pubic Bone Marrow Oedema vs HAGOS

|  |  | Subchondral Pubic Bone Marrow Oedema | |  |  |
| --- | --- | --- | --- | --- | --- |
| HAGOS | Total (N=85) | 0 (N=50) | 1 (N=35) | Difference (95% CI) | p-value (corrected) |
| **Quality of life** |  |  |  |  |  |
| Mean (SD) | 70.5 (28.3) | 81.6 (23.8) | 54.6 (26.9) |  |  |
| Median [Min, Q1, Q3, Max] | 75.0 [10.0, 50.0, 100, 100] | 95.0 [20.0, 62.5, 100, 100] | 55.0 [10.0, 35.0, 70.0, 100] | -40 [-61.3 - -30] | 9.4x10^-5^ |
| **Physical activity** |  |  |  |  |  |
| Mean (SD) | 72.2 (37.0) | 84.8 (29.6) | 54.3 (39.5) |  |  |
| Median [Min, Q1, Q3, Max] | 87.5 [0, 50.0, 100, 100] | 100 [0, 87.5, 100, 100] | 62.5 [0, 18.8, 93.8, 100] | -37.5 [-75 - -25] | 2.3x10^-4^ |
| **Sport and recreation** |  |  |  |  |  |
| Mean (SD) | 74.8 (24.4) | 84.5 (19.1) | 61.0 (24.7) |  |  |
| Median [Min, Q1, Q3, Max] | 81.3 [12.5, 53.1, 100, 100] | 92.2 [25.0, 75.0, 100, 100] | 56.3 [12.5, 43.8, 84.4, 100] | -35.9 [-50 - -18.8] | 9.4x10^-5^ |
| **Activities of daily living** |  |  |  |  |  |
| Mean (SD) | 88.7 (16.7) | 94.1 (12.1) | 81.0 (19.3) |  |  |
| Median [Min, Q1, Q3, Max] | 100 [30.0, 80.0, 100, 100] | 100 [45.0, 95.0, 100, 100] | 85.0 [30.0, 70.0, 100, 100] | - 15 [-35 -15] | 2.8x10^-4^ |
| **Pain** |  |  |  |  |  |
| Mean (SD) | 87.1 (14.7) | 92.8 (9.83) | 78.9 (16.8) |  |  |
| Median [Min, Q1, Q3, Max] | 92.5 [42.5, 77.5, 100, 100] | 97.5 [62.5, 90.0, 100, 100] | 80.0 [42.5, 67.5, 92.5, 100] | -17.5 [-29.3 - -11.3] | 2.0x10^-4^ |
| **Symptoms** |  |  |  |  |  |
| Mean (SD) | 76.0 (19.0) | 83.0 (15.3) | 65.9 (19.4) |  |  |
| Median [Min, Q1, Q3, Max] | 78.6 [25.0, 60.7, 92.9, 100] | 85.7 [35.7, 75.0, 95.5, 100] | 64.3 [25.0, 55.4, 78.6, 100] | -21.4 [-28.6 -5.4] | 2.3x10^-4^ |
| Q1 = 1st quartile Q3 = 3rd quartile | | | | | |

Supplementary Table 4. Capsule/Aponeurosis Tear vs HAGOS

|  |  | Capsule/Aponeurosis Tear | |  |  |
| --- | --- | --- | --- | --- | --- |
| HAGOS | Total (N=85) | 0 (N=74) | 1 (N=11) | Difference (95% CI) | p-value (corrected) |
| **Quality of life** |  |  |  |  |  |
| Mean (SD) | 70.5 (28.3) | 74.1 (27.7) | 46.4 (20.0) |  |  |
| Median [Min, Q1, Q3, Max] | 75.0 [10.0, 50.0, 100, 100] | 85.0 [10.0, 55.0, 100, 100] | 45.0 [10.0, 35.0, 60.0, 80.0] | - 40 [- 60 - -22.5] | 4.2x10^-3^ |
| **Physical activity** |  |  |  |  |  |
| Mean (SD) | 72.2 (37.0) | 75.5 (35.6) | 50.0 (40.3) |  |  |
| Median [Min, Q1, Q3, Max] | 87.5 [0, 50.0, 100, 100] | 100 [0, 53.1, 100, 100] | 75.0 [0, 6.25, 81.3, 100] | -25 [-100 - -12.5] | 1.6x10^-2^ |
| **Sport and recreation** |  |  |  |  |  |
| Mean (SD) | 74.8 (24.4) | 78.8 (23.1) | 47.7 (13.5) |  |  |
| Median [Min, Q1, Q3, Max] | 81.3 [12.5, 53.1, 100, 100] | 84.4 [12.5, 68.8, 100, 100] | 46.9 [25.0, 39.1, 56.3, 65.6] | -37.5 [-51.5 –25.0] | 3.6x10^-4^ |
|  |  |  |  |  |  |
| **Activities of daily living** |  |  |  |  |  |
| Mean (SD) | 88.7 (16.7) | 90.9 (15.8) | 73.6 (14.7) |  |  |
| Median [Min, Q1, Q3, Max] | 100 [30.0, 80.0, 100, 100] | 100 [30.0, 86.3, 100, 100] | 75.0 [50.0, 62.5, 85.0, 95.0] | -25 [-45 - -25] | 2.6x10^-4^ |
| **Pain** |  |  |  |  |  |
| Mean (SD) | 87.1 (14.7) | 88.8 (14.6) | 75.7 (9.62) |  |  |
| Median [Min, Q1, Q3, Max] | 92.5 [42.5, 77.5, 100, 100] | 95.0 [42.5, 82.5, 100, 100] | 72.5 [62.5, 70.0, 83.8, 90.0] | -22.5 [-37.5 - -22.5] | 2.2x10^-3^ |
| **Symptoms** |  |  |  |  |  |
| Mean (SD) | 76.0 (19.0) | 79.2 (17.6) | 54.5 (13.3) |  |  |
| Median [Min, Q1, Q3, Max] | 78.6 [25.0, 60.7, 92.9, 100] | 82.1 [25.0, 67.9, 92.9, 100] | 53.6 [35.7, 46.4, 60.7, 82.1] | -28.6 [-39.3 - - 17.9] | 2.8x10^-4^ |
| Q1 = 1st quartile Q3 = 3rd quartile * Grouped clinical symptoms = one or more of the following; symphysis pubis, adductor insertion, rec abdominis, psoas, inguinal tenderness | | | | | |

Supplementary Table 5. Capsule/Aponeurosis Oedema vs HAGOS

|  |  | Capsule/Aponeurosis Oedema | |  |  |
| --- | --- | --- | --- | --- | --- |
| HAGOS | Total (N=85) | 0 (N=60) | 1 (N=25) | Difference (95% CI) | p-value (corrected) |
| **Quality of life** |  |  |  |  |  |
| Mean (SD) | 70.5 (28.3) | 76.7 (27.5) | 55.6 (25.0) |  |  |
| Median [Min, Q1, Q3, Max] | 75 [10, 50, 100, 100] | 90 [10, 60, 100, 100] | 55 [10, 35, 65, 100] | -35 [-60 - -25] | 2.8x10^-3^ |
| **Physical activity** |  |  |  |  |  |
| Mean (SD) | 72.2 (37) | 77.9 (34.2) | 58.5 (40.6) |  |  |
| Median [Min, Q1, Q3, Max] | 87.5 [0, 50, 100, 100] | 100 [0, 75, 100, 100] | 75 [0, 25, 100, 100] | -25 [-75 - 0] | 5.2x10^-2^ |
| **Sport and recreation** |  |  |  |  |  |
| Mean (SD) | 74.8 (24.4) | 80.1 (23.6) | 62.25 (21.9) |  |  |
| Median [Min, Q1, Q3, Max] | 81.3 [12.5, 53.1, 100, 100] | 90.6 [12.5, 68.0, 100, 100] | 56.25 [25, 43.75, 81.3, 100] | 34.4 [-53.1 - -19.7] | 2.2x10^-3^ |
|  |  |  |  |  |  |
| **Activities of daily living** |  |  |  |  |  |
| Mean (SD) | 88.7 (16.7) | 90.5 (17.5) | 84.4 (14.0) |  |  |
| Median [Min, Q1, Q3, Max] | 100 [30, 80, 100, 100] | 100 [30, 90, 100, 100] | 85 [55, 75, 100, 100] | -15 [-25 - -15] | 1.4x10^-2^ |
| **Pain** |  |  |  |  |  |
| Mean (SD) | 87.1 (14.7) | 89.5 (14.9) | 81.2 (12.8) |  |  |
| Median [Min, Q1, Q3, Max] | 92.5 [42.5, 77.5, 100, 100] | 96.3 [42.5, 85, 100, 100] | 82.5 [62.5, 70, 95, 100] | -13.8 [-23.5 - -2.5] | 3.1x10^-3^ |
| **Symptoms** |  |  |  |  |  |
| Mean (SD) | 76.0 (18.9) | 78.9 (19.1) | 68.9 (17.0) |  |  |
| Median [Min, Q1, Q3, Max] | 78.6 [25, 60.7, 92.9, 100] | 82.1 [25, 64.2, 96.4, 100] | 67.9 [35.7, 57.1, 82.1, 96.4] | -14.3 [-28.6 - -1.8] | 2.3x10^-2^ |
| Q1 = 1st quartile Q3 = 3rd quartile | | | | | |

####

Supplementary Table 6. Cohen’s Kappa for Inter-rater Reliability

| MRI variable | Percent agreement | Cohen's Kappa |
| --- | --- | --- |
| Oedema Main Pubic Body (0-3) (right) | 85.9 | 0.89 |
| Oedema Main Pubic Body (0-3) (left) | 78.8 | 0.85 |
| Subchondral Pubic BMO (0-3) (right) | 70.6 | 0.80 |
| Subchondral Pubic BMO (0-3) (left) | 71.8 | 0.81 |
| Capsule/ADD/RA tear (0/pt=1/ft=2) (right) | 90.6 | 0.89 |
| Capsule/ADD/RA tear (0/pt=1/ft=2) (left) | 90.6 | 0.90 |
| Capsule/ADD/RA oedema (0-3) (right) | 88.2 | 0.90 |
| Capsule/ADD/RA oedema (0-3) (left) | 70.6 | 0.77 |
| Bone spurs, irregularity (0-1) | 100.0 | 1.00 |
| Disc extrusion (0-1) | 98.8 | 0.93 |
| Labral Tear (0/pt=1/ft=2) (right) | 84.7 | 0.83 |
| Labral Tear (0/pt=1/ft=2) (left) | 83.5 | 0.87 |
| Acetabular Subchondral oedema (0-1) (right) | 95.3 | 0.85 |
| Acetabular Subchondral oedema (0-1) (left) | 89.4 | 0.58 |
| Acetabular Cartilage (0/pt=1/ft=2) (right) | 85.9 | 0.74 |
| Acetabular Cartilage (0/pt=1/ft=2) (left) | 87.1 | 0.73 |
| Iliopsoas abnormality (0-1) (right) | 100.0 | 1.00 |
| Iliopsoas abnormality (0-1) (left) | 97.6 | 0.82 |
