# Supplementary figures and images for: MRI findings in athletic groin pain: correlation of imaging with history and examination in symptomatic and asymptomatic athletes
Source: Skeletal Radiol. 2024 Feb 2;54(4):841–50. doi: 10.1007/s00256-024-04603-9 (PMC11845425; doi:10.1007/s00256-024-04603-9)

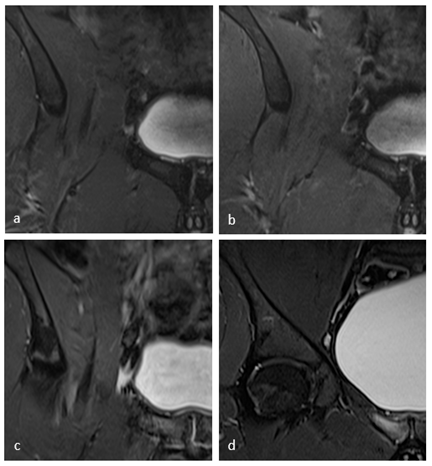

Supplement: Supplementary file 2 — (TIF 232 kb) [file 256_2024_4603_MOESM2_ESM.tif]

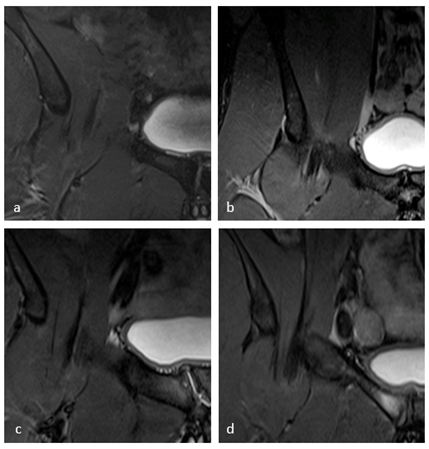

Supplement: Supplementary file 3 — (TIF 257 kb) [file 256_2024_4603_MOESM3_ESM.tif]

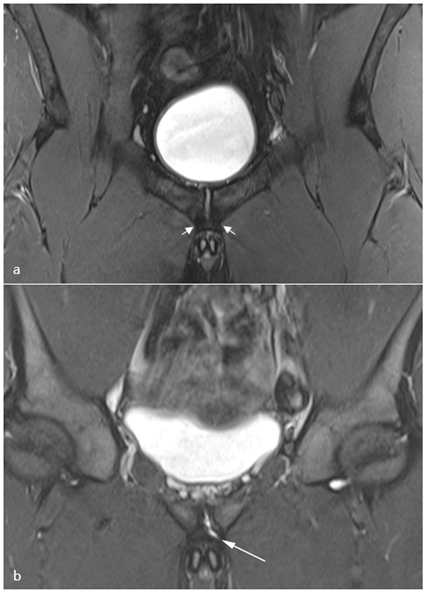

Supplement: Supplementary file 4 — (TIF 371 kb) [file 256_2024_4603_MOESM4_ESM.tif]

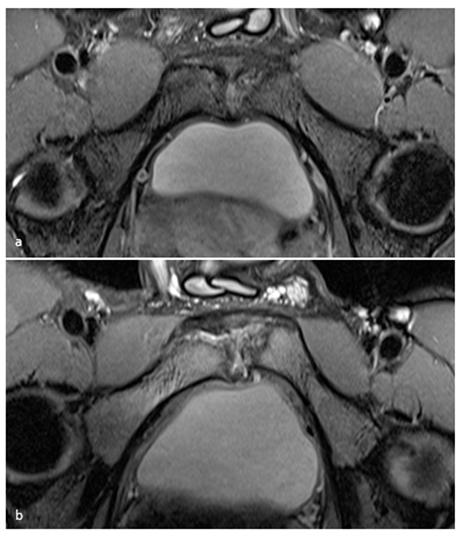

Supplement: Supplementary file 5 — (TIF 368 kb) [file 256_2024_4603_MOESM5_ESM.tif]
